# Superior 125-month outcome through cetuximab in the larynx organ preservation trial DeLOS-II: a single study center’s experience

**DOI:** 10.3389/fonc.2024.1506840

**Published:** 2024-12-24

**Authors:** Gunnar Wichmann, Theresa Wald, Veit Zebralla, Matthaeus Stoehr, Markus Pirlich, Susanne Wiegand, Viktor Kunz, Andreas Dietz

**Affiliations:** ^1^ Clinic for Otorhinolaryngology, University Hospital Leipzig, Leipzig, Germany; ^2^ The Comprehensive Cancer Center Central Germany, Leipzig University Hospital, Leipzig, Germany; ^3^ LIFE - Leipzig Research Center for Civilization Diseases, University of Leipzig, Leipzig, Germany; ^4^ Department of Otorhinolaryngology, Head and Neck Surgery, University Hospital Schleswig-Holstein, Christian-Albrechts-University, Kiel, Germany

**Keywords:** head neck squamous cell carcinoma (HNSCC), locoregional advanced head and neck cancer (LA HNC), larynx cancer, hypopharynx cancer, larynx organ preservation, overall survival, neoadjuvant chemotherapy, cetuximab

## Abstract

**Introduction:**

The larynx organ preservation (LOP) trial DeLOS-II enrolled *n* = 173 patients with advanced laryngeal/hypopharyngeal squamous cell carcinoma (LHSCC) amenable (only curatively resectable) through total laryngectomy (TL) to receive induction chemotherapy (IC) with TPF [docetaxel (T), cisplatin (P), and 5-fluorouracil (F)] (arm A, 85 patients) or additional cetuximab (E) weekly (arm B, 88 patients). Responders with endoscopic estimated tumor surface shrinkage (ETSS) ≥30% after 1 cycle IC (IC-1) received a further two cycles of IC followed by radiotherapy (RT), whereas TL was recommended for non-responders. Arm B failed to show superior 24-month laryngectomy-free survival (LFS) and overall survival (OS), the protocol-specified primary and secondary endpoints. Ten years after the last per-protocol visit, we are interested in the long-term outcome of our clinic’s DeLOS-II patients.

**Methods:**

Our cohort of 52 DeLOS-II patients accrued between 2007 and 2012 included 27 and 25 patients randomized to arms A and B, respectively. F was omitted because of severe toxicity with amendment 2 of the DeLOS-II protocol, leading to 21 and 31 patients receiving TPF and TP IC backbone, respectively. Follow-up data were collected using electronic health records and information from the German Centre for Cancer Registry Data to evaluate long-term LFS and OS in treatment groups.

**Results:**

According to ETSS ≥ 30%, 42 patients (80.8%; 21 and 21 corresponding to 77.8% and 84.0% in arms A and B, respectively) were responders to IC-1 and underwent the LOP attempt. Recommending early TL to non-responders (ETSS < 30%), eight patients (five and three in A and B, respectively) underwent early TL. At 125 months, 22 (eight and 14) patients were alive: 17 (six and 11) with a functioning larynx and five (two and three) without a larynx. Arm B had superior OS (*p* = 0.023). Disease-specific survival (DSS) and tumor-specific survival were not different, whereas non-cancer-related survival (NCRS) was impaired in arm A (*p* = 0.018). Receiving TP or TPF IC did not significantly influence survival. Pairwise comparing OS of patients receiving TP, TPF, TPE, and TPFE revealed a benefit from cetuximab in TPE *vs*. TP (*p* = 0.020).

**Conclusion:**

While the per-protocol DeLOS-II results earlier reported comparable 24-month LFS and OS in arms A and B, our subcohort’s long-term follow-up data demonstrate a superior 125-month outcome in arm B.

## Introduction

The treatment of locally advanced (LA) laryngeal/hypopharyngeal squamous cell carcinoma (LHSCC) is challenging concerning survival and functional outcomes, including talking, swallowing, and breathing. Many tumors are solely completely (R0)–resectable via total laryngectomy (TL) and neck dissection (ND), potentially followed by adjuvant therapy [i.e., postoperative radiotherapy without (PORT) or with concomitant platinum-based chemotherapy (PORCT)]. Concurrent chemoradiation (CRT) with cisplatin (1, 2) and induction chemotherapy with PF (cisplatin and 5-fluorouracil) followed by radiotherapy (3) or TPF (docetaxel or another taxane combined with PF) followed by radiotherapy (4–7) are well-established alternatives to TL + ND + POR(C)T, at least for selected patients with rather small cT3 LHSCC and limited locoregional spread, preferably cN0 or cN1 (8, 9). Induction chemotherapy (IC) + RT and CRT offer the possibility of larynx organ preservation (LOP) by avoiding ablative surgery (1–10). Both IC + RT and CRT are accepted as alternative LOP approaches and are capable of preserving the larynx and its function in a substantial proportion of patients (1–10). In cT4a tumors, however, a surgical approach is recommended, as CRT was found to be associated with decreased survival and high rates of severe late toxicity (11, 12).

Several LOP trials explored IC in patients amenable to surgical treatment with TL (3–7). Inspired by the results of the Bonner trial that demonstrated a benefit from adding cetuximab (Erbitux^®^, E) to radiotherapy (13), the German LOP trial DeLOS-II (14) investigated E added to IC + RT. The objective of DeLOS-II was to evaluate the effect of adding cetuximab (loading dose 400 mg/m^2^ followed by 250 mg/m^2^ weekly for up to 6 months) to IC with docetaxel (T; 75 mg/m^2^ on day 1), cisplatin (P; 75 mg/m^2^ on day 1), and 5-fluorouracil (5-FU; 750 mg/m^2^ on days 1–5; later omitted from the protocol because of severe toxicity). In DeLOS-II, 173 patients were treated with one cycle IC followed by early response evaluation (ERE) using the newly established criterion of endoscopic estimated tumor surface shrinkage (ETSS) ≥30% to discriminate responders and patients with insufficient response (non-responders). Responders with ETSS ≥ 30% additionally received two cycles of IC followed by radiotherapy (RT). Non-responders received the recommendation for TL + ND followed by adjuvant treatment according to the recommendation of the local multidisciplinary tumor board (MDTB). The primary endpoint was 24-month laryngectomy-free survival (LFS), and the secondary endpoints were 24-month overall survival (OS) and 6- and 24-month functional LFS (fLFS), early response after IC-1, toxicity, and complications during and after salvage surgery (14). The primary objective (24-month LFS significantly above 35%) was equally met by both treatment arms (with and without cetuximab). The 24-month OS did not differ significantly between arms (14, 15). This, however, suggested the absence of significantly beneficial effects of additional cetuximab. Within an earlier subgroup analysis conducted after approximately 5 years of follow-up, we also reported slightly different outcomes between patients of arm A *vs.* arm B favoring (15). Now, 12 years after enrolling the last LA-LHSCC patient in DeLOS-II, we evaluated long-term follow-up data of the subgroup of *n* = 52 treated in our University Hospital and compared their outcomes with those achieved by each of the three alternative guideline-conform treatments, TL + ND + PORT, TL + ND + PORCT, and cisplatin-based CRT (16). We found DeLOS-II to be superior regarding the achieved outcome in general and could provide evidence for this superiority based on a comparison of propensity score (PS)-matched cases (16). Here, we provide additional data about the outcome differences among *n* = 52 DeLOS-II patients according to IC backbone, TPF *vs.* TP, the efficacy of cetuximab (arm B *vs.* arm A), and the different outcomes achieved by the four treatment regimens: TPF *vs.* TPFE *vs.* TP *vs.* TPE.

## Methods

Originally, 173 patients were treated according to the DeLOS-II protocol at 22 study centers with the University Hospital Leipzig recruiting 52 patients, the highest number per study center. Detailed information concerning the study protocol is published elsewhere (14).

In brief, the study protocol of DeLOS-II utilized the first cycle of IC (IC-1) consisting of docetaxel (75 mg/m^2^; T) and cisplatin (75 mg/m^2^; P) on day 1 and 5-fluorouracil (750 mg/m^2^; F) on days 1–5 for response evaluation. F was later omitted from the protocol because of severe toxicity. Three weeks after IC-1, an endoscopy under general anesthesia was performed aiming for an estimation of the ETSS. Patients achieving ETSS ≥ 30% were considered responders and received a further two cycles of IC, whereas to poorly responding LHSCC patients with ETSS < 30% or progressing disease, early TL was recommended. Patients randomized into arm B received the identical treatment but additionally E [400 mg/m^2^ loading dose (day 1) followed by 250 mg/m^2^ weekly over 16 weeks in total].

Long-term follow-up data of these 52 patients were collected using electronic health records and information provided by the German Centre for Cancer Registry Data to evaluate 10-year survival including OS, tumor-specific survival (TSS), non-cancer-related survival (NCRS), and LFS. For statistical analyses, SPSS (SPSS version 29, IBM Corporation, Armonk, NY, USA) was used. Univariate analyses of categorical data included Pearson’s chi-squared tests and Fisher’s exact test, while cardinal-metric data were compared using homo- or heteroscedastic Student’s *t*-tests. The Kaplan–Meier cumulative survival plots (17), log-rank tests (18), Cox proportional hazards regression (19) utilizing the conditional logistic regression forward method, and bootstrapping (20) were used to analyze time-to-event data, LFS, OS, disease-specific survival (DSS), TSS, and NCRS. All time-to-event data were calculated from the time of randomization to the event (14, 15). Events were considered death from all causes (OS), death caused by head and neck squamous cell carcinoma (HNSCC) (DSS), death caused by any malignant tumor (HNSCC or cancer of other histology; TSS), or death caused by non-cancer-specific (other than cancer-related) reasons (NCRS), whereas patients alive or without the specified event were right-censored at time of last follow-up. Gaining more importance within the course of follow-up duration, NCRS was reported. Laryngectomy-free survival was calculated from the time of diagnosis to TL. Two-sided *p* < 0.05 was considered significant.

## Results

The characteristics of the 52 DeLOS-II patients treated at the University Hospital Leipzig are shown in **Table 1**, representing 30.1% of the whole study population with 173 patients (14). According to stratification factors in randomization (T4 *vs.* other, N0/N1 *vs.* N2/N3, and larynx *vs.* hypopharynx), arms A and B are not different regarding these covariates and IC backbone TPF *vs.* TP (all *p* ≥ 0.404) and also with respect to age categories, sex, smoking, alcohol consumption, and comorbidities when applying the Charlson score (all *p* ≥ 0.220).

**Table 1:** Characteristics of the 52 DeLOS-II patients treated at the University Hospital Leipzig from 2007 to 2012 in arms A and B of the DeLOS-II trial.

Characteristics		DeLOS-II		Arm A		Arm B		*p*-Value
		(*n* = 52)		(*n* = 27)		(*n* = 25)		
		*n*	(%)	*n*	(%)	*n*	(%)	
Age (years)								
<50		14	(27)	6	(22)	8	(32)	0.594
50–59		23	(44)	14	(52)	9	(36)	
60–69		10	(19)	4	(15)	6	(24)	
≥70		5	(10)	3	(11)	2	(8)	
Sex								
Male		44	(85)	22	(81)	22	(88)	0.515
Female		8	(15)	5	(19)	3	(12)	
Tobacco smoking								
0 pack years		0	(0)	0	(0)	0	(0)	0.999
≤30 pack years		25	(48)	13	(48)	12	(48)	
>30 pack years		27	(52)	14	(52)	13	(52)	
Never		0	(0)	0	(0)	0	(0)	0.220
Former		9	(17)	3	(11)	6	(24)	
Current		43	(83)	24	(89)	19	(76)	
Alcohol consumption								
0	g/d	2	(4)	1	(4)	1	(4)	0.560
1–30	g/d	18	(35)	7	(26)	11	(44)	
31–60	g/d	11	(21)	7	(26)	4	(16)	
>60	g/d	21	(40)	12	(44)	9	(36)	
Tumor location, stage								
Hypopharynx		30	(58)	15	(56)	15	(60)	0.764
Larynx		22	(42)	12	(44)	10	(40)	
T2		6	(12)	3	(11)	3	(12)	0.516
T3		27	(52)	16	(59)	11	(44)	
T4a		19	(37)	8	(30)	11	(44)	
N0		9	(17)	6	(22)	3	(12)	0.404
N1		4	(8)	3	(11)	1	(4)	
N2a		0	(0)	0	(0)	0	(0)	
N2b		20	(38)	11	(41)	9	(36)	
N2c		18	(35)	7	(26)	11	(44)	
N3		1	(2)	0	(0)	1	(4)	
Stage II (UICC)		1	(2)	1	(4)	0	(0)	0.338
Stage III (UICC)		12	(23)	8	(29)	4	(16)	
Stage IVA (UICC)		38	(73)	18	(67)	20	(80)	
Stage IVB (UICC)		1	(2)	0	(0)	1	(4)	
Induction chemotherapy								
TP		31	(60)	16	(59)	15	(60)	0.999
TPF		21	(40)	11	(41)	10	(40)	
ERE after IC-1								
IC-related death		2	(4)	2	(7)	0	–	0.340
Non-responder (ETSS < 30%)		10	(19)	6	(22)	4	(16)	
Responder (ETSS ≥ 30%)		39	(75)	19	(71)	20	(80)	
N/A^a^		1	(2)	0	–	1	(4)	
Total laryngectomy								
No TL		35	(67)	18	(67)	17	(68)	0.676
Early TL		8	(15)	5	(19)	3	(12)	
Late TL		9	(17)	4	(15)	5	(20)	
Radiotherapy								
Yes		49	(94)	24	(89)	25	(100)	0.236
No		3	(6)	3	(11)	0	(0)	
Charlson score								
CS = 0		34	(65)	18	(67)	16	(64)	0.999
CS > 0		18	(35)	9	(33)	9	(36)	

*p*-Values were calculated using Fisher’s exact test and Pearson’s chi-squared test.

T, docetaxel; P, cisplatin; F, 5-FU; ERE, early response evaluation; IC-1, 1st cycle induction chemotherapy; ETSS, endoscopic estimated tumor surface shrinkage; N/A, not available; TL, total laryngectomy.

a Discontinuation of per-protocol treatment due to therapy-related severe adverse event (G3 anaphylactic reaction to Erbitux^®^, treated outside protocol with concurrent chemo-radiotherapy with 300 mg/m^2^ cisplatin and 69.6 Gy).

Overall, the common risk factors including the Charlson comorbidity index were equally distributed among arms A and B, allowing for a reliable comparison of outcome in arm A *vs*. arm B.

Of 52 patients, 25 patients were randomized to the study arm (arm B) with the addition of cetuximab weekly to the regular treatment with TP(F) IC and 27 into arm A (control). A total of 39 patients were considered to be responders to IC-1 according to ETSS ≥ 30% in early response evaluation and consequently selected for the LOP attempt and further IC cycles. Recommending early TL to non-responders with ETSS < 30% after IC-1 (*n* = 6 in arm A and *n* = 4 in arm B), five patients in arm A and three patients in arm B underwent early TL. In five cases, TL within 12 months (i.e., salvage TL) was performed. In total, *n* = 9 patients received late TL within 24 months of follow-up time.

Within 3,769 months of follow-up (mean 72.5, median 63.2, SD 49.8, range 1.6–148 months), 36 patients died (61.5%): 11 patients died from cancer-associated reasons, 21 from non-cancer-related reasons, and four died from another tumor entity.

**Figure 1:**
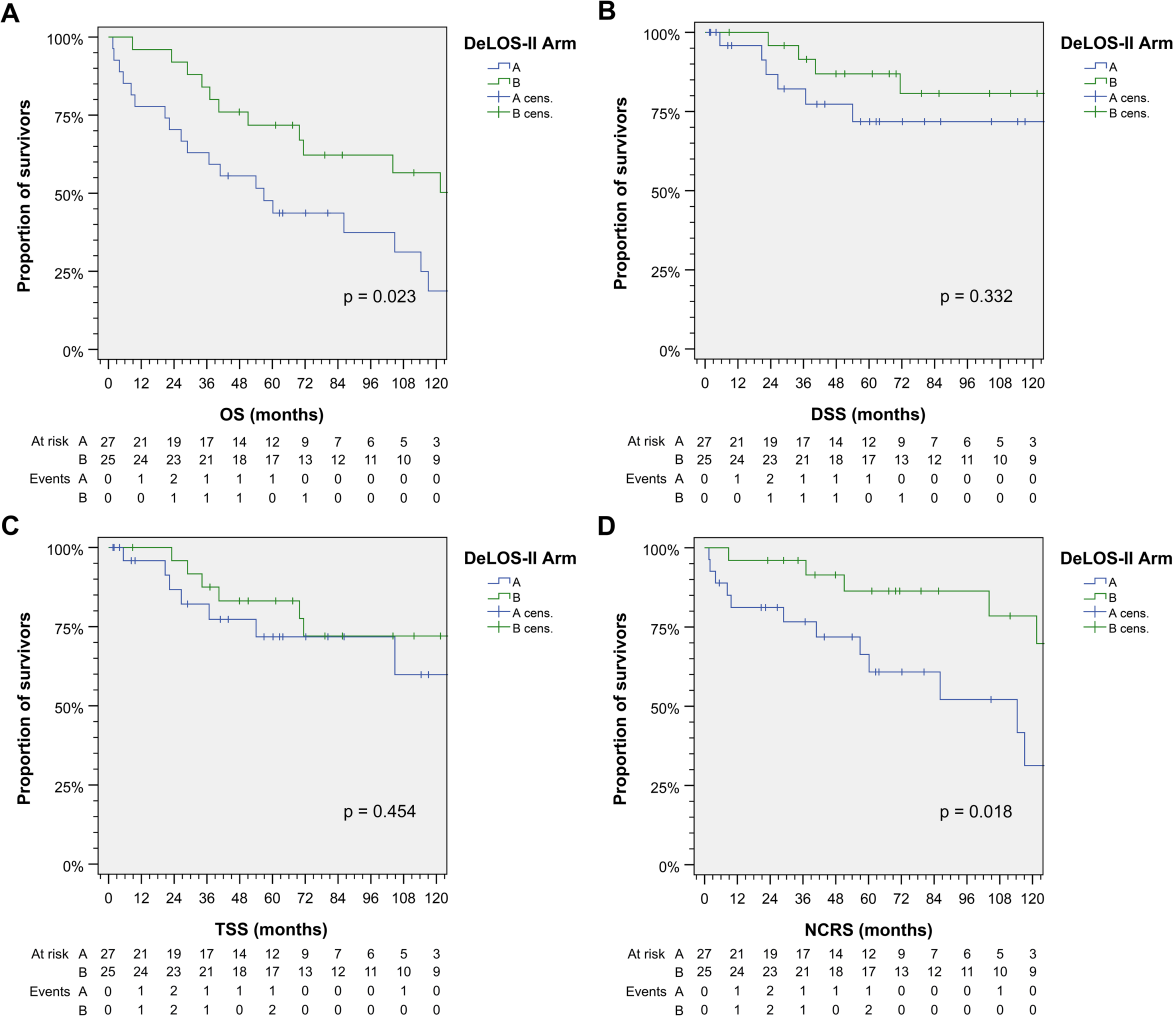
Outcome of DeLOS-II patients according to the treatment arm A *vs.* arm B. Kaplan–Meier cumulative survival plots for **(A)** overall survival (OS), **(B)** disease-specific survival (DSS), **(C)** tumor-specific survival (TSS), and **(D)** non-cancer-related survival (NCRS) among 52 DeLOS-II patients including patients at risk and number of events are shown together with *p*-values from two-sided log-rank tests.

Patients receiving cetuximab (arm B) had improved 125-month OS compared to those without (*p* = 0.023; **Figure 1**). The mean OS in arm A was 64.2 (95% CI 46.1–82.3) months, while the median OS was 56.9 (95% CI 26.1–87.7) months; the mean OS in arm B was 92.2 (95% CI 75.7–108.7) months, while the median OS was not reached. DSS and TSS did not differ significantly between arms. However, NCRS was inferior in arm A (without E; *p* = 0.018). The Kaplan–Meier curves for OS, DSS, TSS, and NCRS for arm A *vs*. arm B are shown in **Figure 1**.

**Figure 2:**
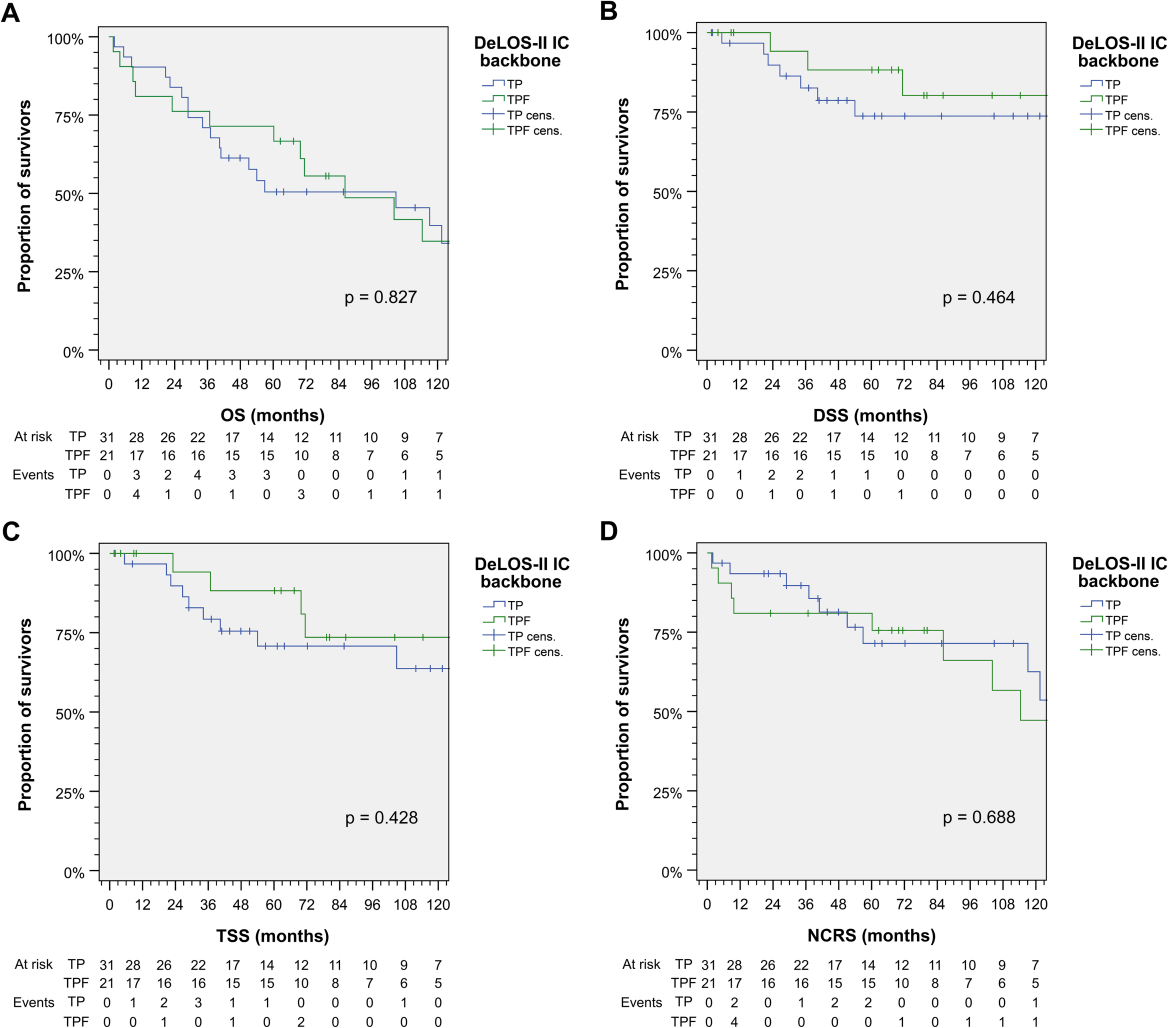
Outcome of DeLOS-II patients according to the induction-chemotherapy backbone TPF *vs.* TP. Kaplan–Meier cumulative survival plots for **(A)** overall survival (OS), **(B)** disease-specific survival (DSS), **(C)** tumor-specific survival (TSS), and **(D)** non-cancer-related survival (NCRS) among 52 DeLOS-II patients including patients at risk and number of events are shown together with *p*-values from two-sided log-rank tests. T, docetaxel; P, cisplatin; F, 5-fluorouracil.

In contrast to significant effects from cetuximab, receiving TP or TPF as chemotherapy backbone did not influence survival significantly (**Figure 2**). As 5-FU caused severe acute toxicity and even therapy-related deaths, it was omitted from the study protocol during the course of the trial. There obviously were differences between TPF and TP according to slight superiority in TSS and DSS, whereas NCRS (based on the attribution of the early deaths as treatment-related) was inferior. Overall, there was no survival benefit detectable from TPF *vs*. TP.

**Figure 3:**
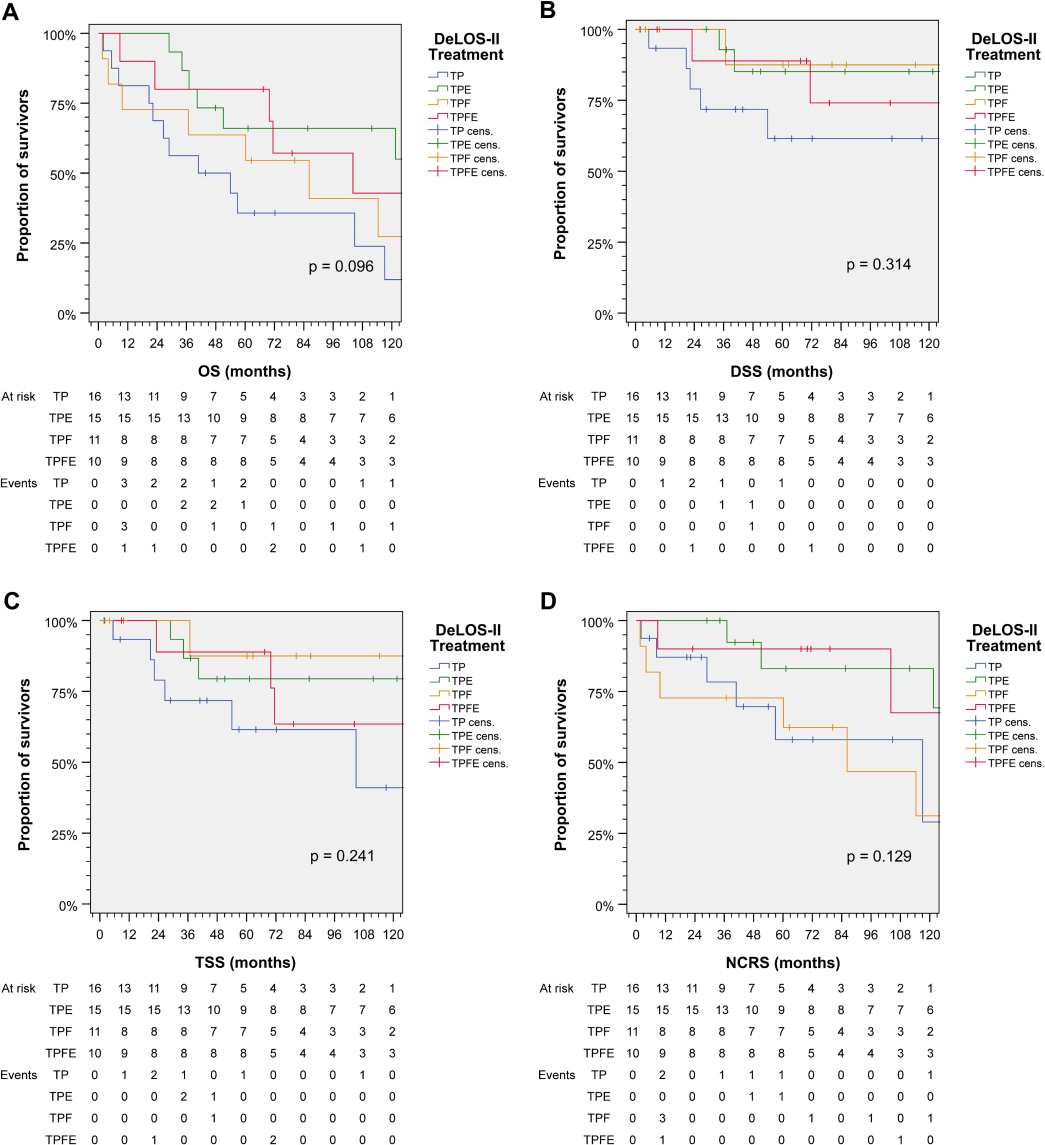
Outcome of DeLOS-II patients according to the exact treatment applied according to treatment arm A (without cetuximab) *vs.* B (receiving cetuximab) and induction-chemotherapy backbone TPF (before amendment 2) *vs.* TP (omission of 5-FU after amendment 2) resulting in TP, TPE, TPF, and TPFE. Kaplan–Meier cumulative survival plots for **(A)** overall survival (OS), **(B)** disease-specific survival (DSS), **(C)** tumor-specific survival (TSS), and **(D)** non-cancer-related survival (NCRS) among 52 DeLOS-II patients including patients at risk and number of events are shown together with *p*-values from two-sided log-rank tests. T, docetaxel; P, cisplatin; F, 5-fluorouracil; E, cetuximab.

**Figure 3** shows cumulative survival plots for the exact treatment (TP *vs*. TPE *vs*. TPF *vs*. TPFE) according to OS, TSS, DSS, and NCRS. Patients receiving one of the four treatment combinations were found to have only insignificantly deviating outcomes regarding DSS, TSS, and NCRS (all *p* ≥ 0.129), whereas OS tended to be different (*p* = 0.096). Indeed, pairwise comparisons revealed that receiving TP resulted in an impaired OS compared to TPE (*p* = 0.020; **Table 2**).

**Table 2:** Comparison of mean [95% confidence interval (95% CI)], median (95% CI), and 75th percentile (95% CI) of overall survival (OS), non-cancer-related survival (NCRS), disease-specific survival (DSS), and tumor-specific survival (TSS) in DeLOS-II patients according to treatment arm and induction-chemotherapy backbone and two-sided *p*-values from log-rank tests for pairwise comparison of Kaplan–Meier cumulative survival curves.

		Mean (95% CI)	Median (95% CI)	75th percentile	TP	TPE	TPF	TPFE
				(95% CI)	*p*-Value	*p*-Value	*p*-Value	*p*-Value
**OS**	TP	58.9 (36.4–81.4)	40.9 (0–86.8)	20.7 (0–44.5)	–	**0.020**	0.425	0.108
	TPE	95.2 (74.3–116.2)	*Not reached*	40.4 (18–62.8)	**0.020**	–	0.205	0.666
	TPF	71.7 (42.4–100.9)	86.2 (16.7–155.7)	9.7 (0–57.2)	0.425	0.205	–	0.464
	TPFE	87.8 (61.4–114.2)	104.1 (25.9–182.3)	69.9 (0–191.5)	0.108	0.666	0.464	–
	*Total*	*77.8 (64.9–90.6)*	*86.2 (39–133.4)*	*28.9 (14.6–43.2)*				
**NCRS**	TP	83.6 (57.5–109.7)	117.1 (28.2–206)	40.9 (0–88.5)	–	0.074	0.846	0.175
	TPE	110.9 (93.8–128.1)	*Not reached*	121.5 (0–277.1)	0.074	–	0.065	0.943
	TPF	77.9 (48.1–107.6)	86.2 (26.3–146.1)	9.7 (0–85.6)	0.846	0.065	–	0.135
	TPFE	108.7 (86.6–130.8)	*Not reached*	104.1 (0–276.3)	0.175	0.943	0.135	–
	*Total*	*96.1 (83.6–108.6)*	*Not reached*	*60.2 (10.6–109.8)*				
**DSS**	TP	87.8 (61.7–114)	*Not reached*	26.6 (0–58.4)	–	0.145	0.199	0.342
	TPE	112 (95.3–128.7)	*Not reached*	*Not reached*	0.145	–	0.893	0.657
	TPF	114 (93.8–134.2)	*Not reached*	*Not reached*	0.199	0.893	–	0.623
	TPFE	105.7 (81.9–129.5)	*Not reached*	71.4 (0–134.5)	0.342	0.657	0.623	–
	*Total*	*103.9 (92.2–115.5)*	*Not reached*	*Not reached*				
**TSS**	TP	83.7 (58.4–108.9)	104.8 (11.8–197.8)	26.6 (0–58.4)	–	0.133	0.117	0.351
	TPE	106.4 (87.7–125.2)	*Not reached*	*Not reached*	0.133	–	0.632	0.605
	TPF	114 (93.8–134.2)	*Not reached*	*Not reached*	0.117	0.632	–	0.398
	TPFE	99.9 (75.9–123.9)	*Not reached*	71.4 (8.3–134.5)	0.351	0.605	0.398	–
	*Total*	*99.4 (87.4–111.4)*	*Not reached*	*69.9 (6.7–133.1)*				

T, docetaxel; P, cisplatin; F, 5-fluorouracil; E, cetuximab. Significant *p* values < 0.05 highlighted bold.

Comparing the mean and median of the four survival parameters OS, DSS, TSS, and NCRS, only TPE emerged as significantly superior to TP. However, we consistently detected numerically longer mean, median, and 75th percentiles for OS in TPFE and TPE (**Table 2**).

Comparing numbers and percentage of patients alive with a functioning larynx, alive after TL, or deceased with a larynx at place or after TL and summarizing data for either LOP or being alive after 125 months (**Table 3**), a consistent superiority of patients treated in arm B is obvious. Despite the low numbers of patients per treatment, a doubled frequency of patients alive at 125 months with a functioning larynx (44%) and 56% survivors in arm B compared to 22% and 30% in arm A point to a benefit gained through addition of cetuximab to IC with TP(F) followed by RT.

**Table 3:** Comparison of outcome of DeLOS-II patients 125 months after treatment indicated including larynx organ preservation and survival status at 125 months.

	Alive with larynx	Alive without larynx	Dead with larynx	Dead without larynx	LOP	Alive at 125 months
	*n* (%)	*n* (%)	*n* (%)	*n* (%)	*n* (%)	*n* (%)
**TP**	3 (18.8)	1 (6.3)	8 (50.0)	4 (25.0)	11 (68.8)	4 (25.0)
**TPE**	7 (46.7)	2 (13.3)	3 (20.0)	3 (20.0)	10 (66.7)	9 (60.0)
**TPF**	3 (27.3)	1 (9.1)	4 (36.4)	3 (27.3)	7 (63.6)	4 (36.6)
**TPFE**	4 (40.0)	1 (10.0)	3 (30.0)	2 (20.0)	7 (70.0)	5 (50.0)
**TP^#^**	10 (32.3)	3 (9.7)	11 (35.5)	7 (22.6)	21 (67.7)	13 (41.9)
**TPF^#^**	7 (33.3)	2 (9.5)	7 (33.3)	5 (23.8)	14 (66.7)	9 (42.9)
**A**	6 (22.2)	2 (7.4)	12 (44.4)	7 (25.9)	18 (66.7)	8 (29.6)
**B**	11 (44.0)	3 (12.0)	6 (24.0)	5 (20.0)	17 (68.0)	14 (56.0)

Shown are number (percentage) among 52 patients (16, 15, 11, and 10 in TP, TPE, TPF, and TPFE groups of treatment, respectively; 21 and 31 in TPF and TP, respectively; and 27 and 25 in arms A and B, respectively).

T, docetaxel; P, cisplatin; F, 5-fluorouracil; E, cetuximab; LOP, larynx organ preservation.

**^#^** Induction-chemotherapy backbone before and after amendment 2 of the DeLOS-II protocol.

## Discussion

Now, available long-term follow-up data of 52 consecutively accrued DeLOS-II patients demonstrate superior OS of patients treated in arm B (*p* = 0.023) predominantly linked to improved NCRS (*p* = 0.018) due to reduced frequency of deaths from other causes, whereas DSS was numerically superior without reaching statistical significance. There were no significant differences in OS, TSS, DSS, and NCRS (all *p* ≥ 0.428) related to the IC backbone, TPF *versus* TP. However, we detected significant survival differences in the orthogonal analysis of arm *vs*. IC backbone with, for example, superior OS achieved by TPE compared to TP (**Tables 2**, **3**).

Altogether, patients presenting with LA LHSCC have an unfavorable outcome. In many cases, TL is the only surgical technique to achieve a complete tumor resection. Because of the need for a tracheostomy, the loss of the natural voice function after TL, and the presumably negative impact on quality of life, LOP attempts are of great interest to ear, nose and throat (ENT) surgeons and oncologists as well as their patients. CRT is a comparable alternative to TL followed by adjuvant therapy, except for patients with cT4a LHSCC (11, 12), as they seem to experience higher recurrence rates when treated with CRT instead of TL + adjuvant therapy. In contrast, within analyses of the DeLOS-II trial data, survival and LOP rates were not inferior for all patients with T4 tumors (14, 15), provided an ETSS ≥ 30% in ERE in week 4 after IC-1 is achieved, and can be recommended also for T4 hypopharynx cancer. As prospective comparisons between the different treatment protocols are rare [except for the Radiation Therapy Oncology Group (RTOG) 91-11 study (1, 2, 21)], a propensity score-matched analysis of patients treated with Op + PORT, Op + PORCT, CRT, or the DeLOS-II protocol showed the non-inferiority (by showing superiority of DELOS-II regarding OS, TSS, and NCRS) of the LOP protocol according to DeLOS-II (16), questioning the non-inferiority of CRT compared to which TL once more, as discussed by Licitra et al. (21, 22).

Within the DeLOS-II trial aiming at LOP, IC followed by RT in responders after response evaluation after IC-1 and TL followed by adjuvant therapy in non-responders were recommended to patients with LA LHSCC. While ERE and achieving ETSS ≥ 30% were highly predictive for cure and LOP (14, 15), even survival of non-responders treated with early TL after IC-1 was superior to that of CRT or TL + PORT and equal to that of TL + PORCT (16). Moreover, ERE after IC-1 helps prevent prolonged administration of an ineffective treatment to the patient and helps prevent salvage surgery with its well-known high complication rates and impossibility of curatively resecting the cancer in inappropriately high frequency (23).

As reported for the per-protocol defined outcome analysis of the whole DeLOS-II trial after 24 months, the study arm with patients receiving cetuximab failed to be superior to the standard arm without cetuximab. The primary endpoint 24-month LFS [both arms with LFS > 35%, arm A (40/85, 47.1%) and arm B (41/88, 46.6%)] and the secondary endpoint 24-month OS (arm A 68.2% and arm B 69.3%) were not different. This, however, could be related to the per-protocol defined 24-month follow-up that could be too short to identify outcome differences and even more long-term outcomes of patients.

In the present study, we provide long-term follow-up data of the patients treated at the University Hospital Leipzig participating in the DeLOS-II study. Surprisingly, after 60 months and especially after 125 months, patients who received cetuximab had significantly better OS than those without. There was no difference between IC with TP and IC with TPF. Analyzing DSS and TSS, patients benefitted from receiving E without statistical significance. In line with this finding, the frequency of relapse, distant metastasis and occurence of other malignancies demonstrate the same (**Supplementary Table S1**, available online). In the long-term, death from other, non-cancer-related causes becomes more impactful and differs significantly in our cohort with benefits for the patients who received cetuximab. In analyses of survival measures, competing risk factors have to be considered. Paying attention to this fact, we analyzed OS by censoring cancer-related deaths in addition to the commonly accepted right-censoring of patients alive at the last follow-up. As age- and senescence-related differences between arms or exact treatment were found to be without significance, the significant differences between arms regarding non-cancer-related survival by only calculating log-rank tests considering death from other causes (not related to cancer) as event appear to be important. Possible confounding of comorbidities was excluded here, as severe comorbidities were an exclusion criterion for DeLOS-II participation (14). Also retrospectively assessing the presence of any comorbidities with a known impact on survival by applying the Charlson score (24, 25), there was no difference between arms (**Table 1**). The causality of the observed association between NCRS and cetuximab remains unclear. Cetuximab enhances the antitumor immune response through the activation of NK cells and facilitates their interaction with tumor cells. Promising, but yet without approval, is the combination of cetuximab and pembrolizumab to overcome cetuximab resistance as shown in *ex vivo* experiments (26). Approved for HNSCC, the anti-epidermal growth factor receptor (anti-EGFR) monoclonal antibody cetuximab (E) is used for system treatment of recurrent or metastatic (R/M) HNSCC alone or in combination with PF [EXTREME (27–29)] or TP [TPExtreme (30) within the TREMPLIN-II study (31)], IC with TPF followed by radiochemotherapy with three more cycles P or bioradiotherapy with E weekly showed no significant differences in LOP and OS. However, because of a high dropout rate of 24% and thus, high selection bias, comparison with other LOP trials is questionable. TREMPLIN-II was conducted to choose an experimental arm to compare to the GORTEC 2000-01 trial (6, 32); as none of the arms was superior to the other, no further study in this regard was attempted.

Several studies on LOP using IC report similar OS rates. Mattei et al. (9) reported in a retrospective study with patients suffering from LA hypopharyngeal cancer only resectable via TL administered with TPF IC followed by RT with or without cisplatin or cetuximab a 5-year OS of 54%. In this study, the T4 category was a negative predictor for OS.

The milestone trial RTOG 91-11 (1, 2) prospectively randomized patients into different treatment regimens consisting of IC + RT, CRT, or RT alone between 1992 and 2000. They report a 5-year OS of 58.1% and a 10-year OS of 38.8% (2). LOP was 67.5% after 10-year follow-up (FU), matching exactly the above-reported LOP data in arms A and B in **Table 3**.

Pointreau et al. (6, 32) found 60-month OS of 51% and 120-month OS of 30% comparing survival rates of 213 patients administered with three cycles of IC with either TPF or PF followed by radio(chemo)therapy for responders or laryngectomy for non-responders. The authors recommend IC with TPF, as there were higher rates of LOP compared to PF only. This is in line with the findings of Vermorken et al., who described a survival benefit when applying TPF instead of PF only in patients with unresectable LHSCC (33).

However, the 60-month OS rate in the cohort of 52 DeLOS-II patients with 71.8% in arm B administered with TPE or TPFE was clearly superior. After 125-month FU, the OS rate was still 56.0%, differing significantly from the 29.6% in the TP/TPF group (arm A; **Table 3**) and according to the log-rank test (*p* = 0.023; **Figure 1A**), clearly surpassing the survival data of comparable studies mentioned above (1, 2, 22, 23, 31, 33).

The long-term outcomes of the complete cohort of DeLOS-II patients would have been desirable but unfortunately could not be obtained. Reflecting 30.1% (52/173) of the complete study cohort, our results still suggest a potential additional benefit when adding cetuximab to the IC, the RT, and 6 months in total to the DeLOS-II protocol. Further studies are needed, preferably randomized controlled trials (RCTs), to find out and evaluate the best treatment protocol for LOP attempts only via TL R0-resectable LHSCC patients. Other agents promising a treatment benefit should be examined. For instance, pembrolizumab is known and approved for the treatment of R/M HNSCC because of its antitumor effect that is enriched in HNSCC patients with a certain level of PD-L1 expression in their tumor. The tumor proportion score (TPS) is calculated as the percentage of cells staining positive for PD-L1 in immunohistochemistry among tumor cells, and the combined positive score (CPS) also considers immune cells (34); after KEYNOTE-048 (35), pembrolizumab is approved for R/M HNSCC with CPS ≥ 1 (which of course includes TPS ≥ 50%). KEYNOTE-689 investigates in LA HNSCC with CPS ≥ 1 the neoadjuvant treatment with two cycles of pembrolizumab upfront surgery followed by a further 15 cycles of pembrolizumab during adjuvant radiotherapy ± cisplatin (36). The European Larynx Organ Preservation Study [ELOS (37)] investigates the effect of pembrolizumab when added to IC with TP followed by RT according to the DeLOS-II protocol. It remains to be shown if replacing cetuximab with pembrolizumab enables higher LOP and OS rates.

One strength of our study is the high number of participants per subgroup accrued and treated within a prospectively designed RCT at a single University Hospital. This led to 1) homogeneity in decision-making for participation in the LOP trial according to uniform per-protocol defined inclusion and exclusion criteria, 2) random assignment to pre-defined treatment groups while stratifying according to characteristics/covariates with the highest impact on outcome, 3) response evaluation according to the protocol-defined ERE criteria by a well-trained single ENT surgeon familiar with LOP, 4) regular follow-up of patients according to the National Comprehensive Cancer Network (NCCN) and national guidelines, 5) regular monitoring of organ function and health-related quality of life utilizing patient-reported outcome measures (PROM) using OncoFunction (38, 39) to detect early signs of functional impairment, and 6) high quality of documentation attributed to study patients. In addition to these strengths, there are limitations that have to be considered. Our results were achieved in (according to inclusion and exclusion criteria) highly selected patients suffering from a rare disease. Despite allowing for the detection of significant differences between groups, the number of patients in each of the treatment groups was rather low with <30 patients in each subgroup analyzed. This increases the possibility of random effects and, in particular, increases the false-positive reporting probability (FPRP) of (additionally in tendency rather to high) effect estimates, as small samples favor the overrepresentation of extreme distributions. Rather few patients per treatment may have caused a positive reporting bias that we cannot exclude. An increased FPRP could even more be expected, as the exploratory analyses of outcome measures were not pre-planned/scheduled within the DeLOS-II protocol and especially were not considered in the power calculation for the DeLOS-II trial. Because of the rather low accrual of patients in the other 22 DeLOS-II study centers, we accrued the highest number of DeLOS-II patients treated in our center and had the unique opportunity to assess long-term outcomes with sufficiently high numbers of patients per treatment in a unique environment. Our patients were treated in a certified tertiary tumor center of excellence, the Comprehensive Cancer Center Central Germany (CCCG). The CCCG has an infrastructure allowing for high-quality patient-centered workflows with professionalized interaction of all disciplines involved in diagnostic, treatment, and follow-up monitoring of head and neck cancer patients, patients who are always a special group of LA LHSCC patients with special and often unmet needs. Assessing these special needs (38, 39) during prolonged follow-up and addressing them adequately may have contributed to the overall good outcome. Building such infrastructure requires time and dedicated professionals in each of the clinics and institutes involved but especially dedicated consultation hours regularly utilizing the adequate tools (38, 39). Such an environment could hardly be immediately established in any clinic. Therefore, the results reported here may not be representative of results that can be easily achieved anywhere by *ad hoc* starting the LOP approach for every patient with LA LHSCC amenable for TL by applying (potentially without the availability of the infrastructure required) the treatment according to the DeLOS-II protocol utilizing TPE IC. The ERE after IC-1 assessing ETSS for decision-making for treatment with either TL or further IC followed by radiotherapy requires familiarity with the approach and hence an adequate training.

However, the superior survival in DeLOS-II patients treated with cetuximab and especially TPE points to a so far not reported benefit from cetuximab in LOP approaches, which deserves further investigation, for instance, in a LOP trial combining TPE IC with blockade of the PD-1:PD-L1 immune checkpoint by, for example, pembrolizumab or another monoclonal anti-PD-1 antibody. The LOP approach may additionally benefit from molecular and genetic analyses (40–42). Even a tailored approach according to a comprehensive predictive classifier integrating clinical, molecular, and multiomics data for personalized treatment decisions enhancing response to IC, to achieve LOP in a further increased frequency of patients (43), has the potential to result in long-term survival with good health-related quality of life, altogether the ultimate goals of preventive, predictive, and personalized medicine.

## Data Availability

The raw data supporting the conclusions of this article will be made available by the authors, without undue reservation.
