# Supplementary material for: Superior 125-month outcome through cetuximab in the larynx organ preservation trial DeLOS-II: a single study center’s experience
Source: Front Oncol. 2024 Dec 24;14:1506840. doi: 10.3389/fonc.2024.1506840 (PMC11703891; doi:10.3389/fonc.2024.1506840)
Supplement: Supplementary file 1 [file Table1.pdf]

Table S1. Numbers and percentage of cancer-specific events occurring within 125 months of follow-up in *n*=52 patients treated within the DeLOS-II larynx organ preservation trial at one university hospital according to type of induction chemotherapy received. Based on small numbers of events among small subgroups of patients which received either TP, TPF, TPE or TPFE neither significant nor otherwise systematic differences between cancer-specific events were obvious.

**Events within 125 months**			**Arm A**				**Arm B**			
	**Total**	**(in *n* =52)**	**TP**	**(in *n* =16)**	**TPF**	**(in *n* =11)**	**TPE**	**(in *n* =15)**	**TPFE**	**(in *n* =10)**
Locoregional recurrence	10	(19.2%)	1	(18.8%)	1	(18.2%)	2	(26.7%)	1	(10%)
Local relapse	5	(9.6%)	3	(6.3%)	2	(9.1%)	4	(13.3%)	1	(10%)
Nodal relapse	2	(3.8%)	1	(6.3%)	0	--	1	(6.7%)	0	--
Distant metastasis	2	(3.8%)	2	(12.5%)	0	--	0	--	0	--
Second HNSCC	4	(7.7%)	0	--	0	--	3	(20%)	1	(10%)
Other cancer entity	4	(7.7%)	2	(12.5%)	1	(9.1%)	0	--	1	(10%)

TP – docetaxel + cisplatin; TPF – docetaxel + cisplatin + 5-fluorouracil; TPE – docetaxel + cisplatin + cetuximab; TPFE – docetaxel + cisplatin + 5-fluorouracil + cetuximab. The first relapse or other cancer-specific event only is shown. Local or nodal relapse were jointly considered and counted as locoregional recurrence independent from being diagnosed solely or simultaneously.
